# Sclerostin, vascular risk factors, and brain atrophy in excessive drinkers

**DOI:** 10.3389/fnhum.2023.1084756

**Published:** 2023-02-21

**Authors:** Candelaria Martín-González, Ana María Godoy-Reyes, Pedro Abreu-González, Camino María Fernández-Rodríguez, Esther Martín-Ponce, María José Sánchez-Pérez, Julio César Alvisa-Negrín, Melchor Rodríguez-Gaspar, Emilio González-Reimers

**Affiliations:** ^1^Departamento de Medicina Interna, Universidad de La Laguna, Servicio de Medicina Interna, Hospital Universitario de Canarias, San Cristóbal de La Laguna, Spain; ^2^Departamento de Ciencias Médicas Básicas, Unidad de Fisiología, Universidad de La Laguna, San Cristóbal de La Laguna, Spain

**Keywords:** brain atrophy, ethanol, alcoholism, vascular calcification, sclerostin

## Abstract

**Objective:**

Heavy alcohol consumption causes several organic complications, including vessel wall calcification. Vascular damage may be involved in the development of brain atrophy and cognitive impairment. Recently, sclerostin (whose levels may be altered in alcoholics) has emerged as a major vascular risk factor. The objective of the present study is to analyze the prevalence of vascular calcifications in alcoholics, and the relationships of these lesions with brain atrophy, as well as the role of sclerostin on these alterations.

**Patients and methods:**

A total of 299 heavy drinkers and 32 controls were included. Patients underwent cranial computed tomography, and several indices related to brain atrophy were calculated. In addition, patients and controls underwent plain radiography and were evaluated for the presence or absence of vascular calcium deposits, cardiovascular risk factors, liver function, alcohol intake, serum sclerostin, and routine laboratory variables.

**Results:**

A total of 145 (48.47%) patients showed vascular calcium deposits, a proportion significantly higher than that observed in controls (χ^2^ = 16.31; *p* < 0.001). Vascular calcium deposits were associated with age (*t* = 6.57; *p* < 0.001), hypertension (*t* = 5.49; *p* < 0.001), daily ethanol ingestion (*Z* = 2.18; *p* = 0.029), duration of alcohol consumption (*Z* = 3.03; *p* = 0.002), obesity (χ^2^ = 4.65; *p* = 0.031), total cholesterol (*Z* = 2.04; *p* = 0.041), triglycerides (*Z* = 2.05; *p* = 0.04), and sclerostin levels (*Z* = 2.64; *p* = 0.008). Calcium deposits were significantly related to Bifrontal index (*Z* = 2.20; *p* = 0.028) and Evans index (*Z* = 2.25; *p* = 0.025). Serum sclerostin levels were related to subcortical brain atrophy, assessed by cella media index (*Z* = 2.43; *p* = 0.015) and Huckmann index (ρ = 0.204; *p* = 0.024). Logistic regression analyses disclosed that sclerostin was the only variable independently related to brain atrophy assessed by altered cella media index. Sclerostin was also related to the presence of vascular calcifications, although this relationship was displaced by age if this variable was also included.

**Conclusion:**

Prevalence of vascular calcification in alcoholics is very high. Vascular calcium deposits are related to brain atrophy. Serum sclerostin is strongly related to brain shrinkage and also shows a significant relationship with vascular calcifications, only displaced by advanced age.

## Introduction

Ethanol is a toxic compound for human beings. Although, classically, the boundary of the amount of ethanol consumption associated with organic complications was situated at about 50 g/day for men (21 drinks a week) and 30 g/day for women (Reid et al., 1999), more recent epidemiological studies show that drinking more than 100 g/week may shorten lifespan (Wood et al., 2018), and consumption of more than 30 g/day among men or 5–15 g/day among women may be associated with increased cancer risk (Cao et al., 2015). Therefore, the definition of the safe alcohol consumption limits is an issue subjected to debate.

Heavy drinkers develop many important, life-threatening complications. Although liver, cancer, or pancreatic disease constitute outstanding alcohol-related disorders, alcoholic cardiomyopathy, osteosarcopenia/osteosarcopenic adiposity, or brain affectation are very commonly associated with ethanol consumption and importantly contribute to morbidity and mortality of these patients.

Brain damage may severely impair the quality of life of alcoholics. There is general agreement regarding the deleterious influence of heavy ethanol consumption on brain structure and function, but some controversy exists in relation to the effect of light-to moderate ethanol consumption on brain alterations (Ridley et al., 2013; Rehm et al., 2019; Peng et al., 2020). This controversy may be due to the presence of many confounding factors associated with alcoholism, that add to the widespread direct or indirect changes caused by ethanol on metabolic pathways potentially involved in adequate brain function. In addition to the direct inhibitory effects of ethanol on neurogenesis, proinflammatory cytokines and oxidative stress may cause neuroinflammation and neurodegeneration (Crews and Nixon, 2009; Qin and Crews, 2012). Repeated microtrauma associated with the bizarre style of life of many alcoholics, prone to aggression and violence may also contribute (Harris et al., 2019). Altered nutritional status (Romero-Acevedo et al., 2019), or several micronutrient deficiencies also probably play a role (González-Reimers et al., 2014), especially thiamine deficiency (Topiwala and Ebmeier, 2018).

One of the hallmarks of the alcoholic brain shrinkage and cognitive impairment is the potential recovery after alcohol cessation (Pfefferbaum et al., 1995), something that supports the idea of a transient effect of ethanol on some metabolic pathways, that recover their function after alcohol withdrawal. However, in many patients brain functional and/or morphological recovery may be incomplete despite alcohol cessation (Ridley et al., 2013; de La Monte and Kril, 2014), an observation possibly related to the existence of an already established organic damage. In this sense, some authors have reported that heavy alcohol consumption increases τ phosphorylation and β amyloid accumulation [features of Alzheimer disease, (Peng et al., 2020)] but a direct correlation between alcohol consumption and Alzheimer disease has not been described (Ehrlich et al., 2012).

In heavy alcoholics, in addition to the presence of lesions like those observed in Alzheimer disease, brain lesions derived from vascular alterations may be also present. Vessel wall calcifications constitute a hallmark of vascular damage and are associated to increased vascular risk (Rennenberg et al., 2009). Vessel wall calcifications are frequent in heavy drinkers (Shi et al., 2020), as pointed out in previous studies. For instance, Pletcher et al. (2005) report a clear-cut, independent association among coronary artery calcification and ethanol consumption. Oros et al. (2012) have clearly shown that ethanol promotes vascular smooth muscle cells calcification and transition of these cells to osteoblastic-like cells, providing a strong support to the finding of radiologically detectable vascular calcification in excessive drinkers, independently of the concomitant presence of hypertension. In a study performed in Korea, there was a parallel increase in the incidence of coronary artery calcification and ethanol ingestion, as well as a relationship between ethanol ingestion and hypertension (Yun et al., 2017). Indeed, increased prevalence of hypertension has been reported in alcoholics (Saunders, 1987; van Leer et al., 1994; Fuchs et al., 2001; Fuchs and Fuchs, 2021). Hypertension in alcoholics may develop either by the effects of ethanol by itself (Marmot et al., 1994) or through other factors associated with alcoholism, such as tobacco consumption, gender, or age. It is well known that hypertension is associated with vascular damage, smooth muscle cell remodeling, and vessel wall calcification (Shi et al., 2020). Therefore, in alcoholic patients, vessel wall calcification may be related both to the effect of ethanol by itself and/or to associated hypertension.

The chronic “smoldering” inflammatory status associated with heavy ethanol drinking surely plays a major role on the development of vascular calcifications, together with the simultaneous presence of other risk factors, such as diabetes, or dyslipidemia. Moreover, the multisystemic effects of ethanol may alter the expression and/or functional activity of diverse compounds involved in vascular damage. Recently, the role of sclerostin as a new vascular risk factor has gained attention (Catalano et al., 2020). Sclerostin is a member of the so called osteokines, i.e., bone derived cytokines able to exert a variety of functions in the intermediate metabolism (Kirk et al., 2020). In the last decade several authors have analyzed the role of this molecule on vascular calcifications, observing in *in vitro* studies that sclerostin was involved in medial vascular smooth muscle cells calcification (Zhu et al., 2011), in the formation of the atherosclerotic plaque, as shown by Leto et al. (2019) in 46 patients undergoing carotid endarterectomy, and also in the calcification of the aortic valve (Koos et al., 2013). In other clinical studies serum sclerostin levels were related to vascular calcification in 51 patients with end-stage renal disease (Li et al., 2019). Pelletier et al. (2015) also found a marked association between high sclerostin levels and aortic calcification in 53 patients with chronic kidney disease. Some studies have analyzed the behavior of sclerostin among patients with alcoholism or liver disease -although with conflicting reported results (González-Reimers et al., 2013; Wakolbinger et al., 2020; Jadzic et al., 2022; Martín González et al., 2022) but the relationship of sclerostin with vascular changes and/or brain alterations among alcoholics has received little attention.

Based on these facts, in the present study we want to analyze the prevalence of vascular calcifications and the relationship of these vascular lesions with computed tomography (CT)-assessed brain alterations in alcoholics, and to explore the relationship of the osteokine sclerostin with vascular calcification and brain damage in a subset of these patients.

## Patients and methods

**FIGURE 1:**
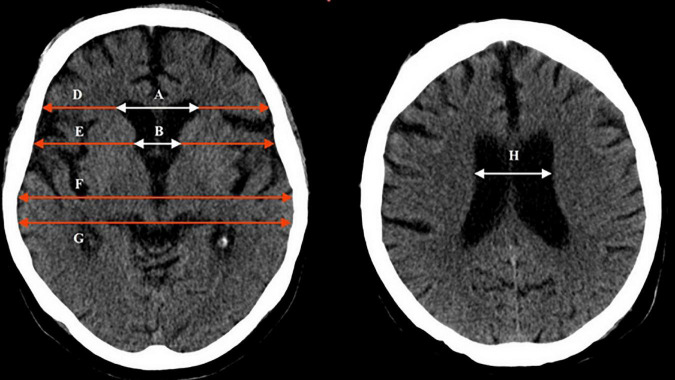
Brain computed tomography, indicating the different indices used in this study and how they were calculated: Bifrontal index = maximum width of frontal horns/skull width at the same level = A/D. Evans index = maximum width of frontal horns/skull width at the level of the third ventricle = A/F. Bicaudate index = minimum width of lateral ventricles/skull width at the same level = B/E. Ventricular index = minimum width of lateral ventricles/maximum width of frontal horns = B/A. Cella media index = maximum width of the skull/width of lateral ventricles = G/H.

In this observational study we included 299 patients consecutively admitted *via* emergency room to the Internal Medicine Unit of our Hospital due to organic problems related to alcohol consumption. The sample included 271 men and 28 women, who underwent cranial CT at admission (in most cases by withdrawal syndrome), and calculation of several classic indices related to brain shrinkage (Huckmann, Evans, bifrontal, ventricular, bicaudate, cella media; Figure 1). The only selection criteria required were chronic consumption of a daily amount of at least 80 g pure ethanol (men) or 40 g (women) for at least the last 5 years before admission; and the clinical indication of a brain CT study (mainly coma, seizures, withdrawal syndrome, traumatism). Patients with meningitis/meningoencephalitis, intracranial bleeding, brain abscess or tumor, were excluded. The last 122 patients were included in a prospective study devoted to analyzing the behavior of sclerostin in relation with brain alterations and vascular calcifications.

All the patients were heavy drinkers. The absolute amount of alcohol consumed was estimated by direct inquiry, both to the patients and close relatives, recording type of beverage(s) and daily amount ingested to calculate amount (in g) of ethanol consumed as: degree of beverage (in%) × beverage volume × 0.8 (alcohol density). Included patients drank a median daily ethanol amount of 197 g [interquartile range (IQR) = 100–250 g] during 31 (IQR = 24–40) years. Patients who consumed any other drug besides tobacco (smoked by 202 patients) were not included in this study. All the patients underwent a complete laboratory evaluation. Body mass index (BMI) was calculated as weight (kg)/height (m2).

A plain thoracic X-ray film was performed to all these patients. In 295 cases the presence or not of calcium deposits in the aortic arch was assessed (in the remaining 4 cases poor X-ray quality precluded accurate evaluation).

Plain X-ray film was also performed to 32 sanitary workers, drinkers of less than 10 g ethanol/day, with similar sex distribution (29 men, 3 women; χ^2^ = 0; *p* = 1) and age (53.25 ± 11.11 years; *t* = 1.93; *p* = 0.06) than the alcoholic patients (57.35 ± 11.40) years. The individuals belonging to the control group were randomly selected among hospital workers, previous informed consent. The only criteria for selection were to be either teetotalers or occasional drinkers of less than 10 g/day; having an approximate age/sex distribution to that of the patients; and having not suffered any brain illness, intervention, or traumatic event.

In addition to complete clinical evaluation, patients also underwent abdominal ultrasound (US) examination. The presence of splenomegaly and/or portal dilatation and a heterogeneous liver structure and irregular shape, together with altered levels either of albumin, bilirubin, or prothrombin activity, served us to classify the patients as cirrhotics, a condition fulfilled by 126 out of the 299 patients, whereas the remaining 173 were classified as non-cirrhotics. We also recorded the presence or not of liver steatosis (that was observed in 128 patients), based on US examination. To achieve a global assessment of liver function, we applied the Child-Pugh score to the whole sample, despite being aware that this score was initially designed as a prognostic tool only for cirrhotics. Child score is based on the alteration of the following variables: serum albumin, bilirubin, prothrombin activity, and presence/severity of ascites and/or encephalopathy (Child and Turcotte, 1964; Pugh et al., 1973). The presence of hypertension (previous or current diagnosis) or diabetes were also recorded.

### Laboratory assessment

All the patients underwent complete routine laboratory analysis. Blood samples were taken at 8.00 am in fasting conditions, in order to determine serum levels of variables related to ethanol consumption such as gamma glutamyl transferase (GGT) and mean corpuscular volume (MCV); liver function variables such as bilirubin, albumin, and prothrombin activity; serum creatinine; and variables related to metabolic syndrome, such as total, LDL and HDL cholesterol, triglycerides, uric acid, and glycated hemoglobin. Serum sclerostin was determined to 122 patients and 31 controls by ELISA method, using a commercial kit purchased from Thermo Scientific Laboratories (Thermo Fisher Scientific Co., Waltham, MA, USA). The calibration curve of ELISA was set 0–10,000 pg/ml. The assay was evaluated with a 4PL algorithm. The correlation analysis between absorbance units (AU) and standards was 0.9945. The λ max of the analysis was established at 450 nm, using a microplate spectrophotometer reader (Spectra MAX-190, Molecular Devices, Sunnyvale, CA, USA). The lower limit of detection (zero + 2 SD) of this assay was 12 pg/ml. Intra and inter-assay coefficients of variation (CV) were 4.32% and 5.18%, respectively. The final serum concentration of sclerostin was expressed in pmol/L (conversion factor: 1 pg/ml = 0.044 pmol/L, molecular weight = 22.5 kDa).

### Statistical analysis

The Kolmogorov–Smirnov test was used to test for normal or Gaussian distribution, a condition not fulfilled by several variables. Therefore, non-parametric tests, such as Mann–Whitney’s U test and Kruskal–Wallis test and Spearman’s correlation analysis were used to analyze differences or correlations among non-parametric variables. When the variables subjected to analysis showed a normal distribution, Student’s *t* test, variance analysis and Pearson’s correlation analysis were used. Stepwise logistic regression analyses (dichotomizing the selected variables according to medians) were used to discern if a given result obtained in the univariate analyses was independent of confounding factors. Multiple linear regression analyses were also used to disclose the confounding effect of age (or other continuous variables) on significant results observed in the univariate analyses. In addition, the ability of sclerostin as a diagnostic marker of vascular damage and/or brain atrophy was also explored using ROC curves analysis. Considering that hypertension is a well-known factor involved in vascular damage, the sensitivity and specificity of sclerostin levels over the median in the diagnosis of vascular calcification or brain shrinkage (assessed by ROC curves) were tested both in the whole group and in the non-hypertensive group. All these analyses were performed with the SPSS program (Chicago, IL, USA).

The study protocol was approved by the local ethical committee of our Hospital (number 2017/50) and conforms to the ethical guidelines of the 1975 Declaration of Helsinki. All the patients gave their written informed consent.

## Results

### Vascular calcifications

One hundred and forty-five patients (48.47%) showed calcium deposits in the thorax plain X-ray films, a proportion by far higher than that observed among controls (χ^2^ = 16.31; *p* < 0.001, Table 1). Age was significantly higher among patients with vascular calcifications (*t* = 6.57; *p* < 0.001).

**TABLE 1:** Some biological features in patients with or without vascular calcium deposits and with or without hypertension.

	Vascular calcium deposits			Hypertension		
	Yes (*n* = 143)	No (*n* = 152)	T (Z); p	Yes (*n* = 117)	No (*n* = 177)	T (Z); p
Age (years)	61.62 ± 10.89	53.50 ± 10.34	*T* = 6.57; *p* < 0.001	61.80 ± 11.50	54.69 ± 10.43	*T* = 5,49; *p* < 0.001
Daily ethanol ingestión (g)	191.32 ± 133.40 156 (100–225)	200.58 ± 84.36 200 (132–274)	*Z* = 2.18; *p* = 0.029	197.63 ± 93.44 200 (120–250)	193.46 ± 120.94 180 (100–250)	*Z* = 0.96; NS
Duration of alcohol consumption (years)	33 ± 12 30 (26–40)	29 ± 11 30 (20–35)	*Z* = 3.03; *p* = 0.002	34 ± 12 30 (25–40)	29 ± 11 30 (20–35)	*Z* = 3.21; *p* = 0.001
Tobacco consumption	103/141	96/152	X2 = 2.84; NS	77/115	123/177	X2 = 0.11; NS
Packets/year index	48.86 ± 33.28 40 (30–60)	41.70 ± 25.55 36 (25–50)	*Z* = 1.75; *p* = 0.08	40.40 ± 27.77 35 (25–43)	48.15 ± 30.62 40 (30–60)	*Z* = 2.14; *p* = 0.033
Obesity (BMI > 30 kg/m^2^)	26/113	15/128	X2 = 4.65; *p* = 0.031	27/93	14/147	X2 = 13.96; *p* < 0.001
Liver cirrhosis	57/143	67/152	X2 = 0.38; NS	47/117	75/177	X2 = 0.07; NS
Prothrombin activity (%)	78.80 ± 21.11 85 (64–100)	77.43 ± 21.43 85 (57–100)	*Z* = 0.48; NS	78.41 ± 20.19 85 (65–98)	77.65 ± 21.99 84 (57–100)	*Z* = 0.04; NS
Serum bilirubin (mg/dl)	2.34 ± 3.32 1.0 (1.0–2.3)	2.96 ± 4.55 1.4 (1.0–3.4)	*Z* = 0.84; NS	2.53 ± 3.60 1.1 (1.0 –2.30)	2.75 ± 4.27 1.2 (1.0–3.3)	*Z* = 0.45; NS
Serum albumin (g/dL)	3.48 ± 0.73 3.5 (3.1–3.9)	3.53 ± 0.75 3.7 (2.9–4.1)	*Z* = 1.04; NS	3.61 ± 0.77 3.7 (3.1–4.2)	3.43 ± 0.72 3.5 (3.0–4.0)	*Z* = 1.66; NS
MCV (fL)	99.73 ± 9.31 100.0 (95.1–104.6)	99.36 ± 8.28 100.2 (94.6–104.6)	*Z* = 0.01; NS	98.64 ± 8.40 99,1 (93.8–103.6)	100.35 ± 9.01 100.6 (95.8–105.7)	*Z* = 2.00; *p* = 0.046
Platelet count (x10^3^/mm^3^)	198505.59 ± 111806.46 180000 (117000–267000)	178532.89 ± 134912.77 142000 (87000–234000)	*Z* = 2.36; *p* = 0.018	195341.88 ± 114401.23 176000 (108000–243000)	184634.46 ± 130445.28 150000 (94500–243000)	*Z* = 1.33; NS
Serum GGT (U/L)	237.36 ± 332.31 113.0 (50.0–301.0)	309.66 ± 473.17 157.5 (77.3–362.5)	*Z* = 2.56; *p* = 0.011	289.59 ± 398.06 128.0 (62.5–361.5)	256.36 ± 408.38 151.0 (66.5–319.0)	*Z* = 0.26; NS
Diabetes mellitus	31/143	42/150	X2 = 1.24; NS	47/117	26/177	X2 = 23.16; *p* < 0.001
Liver steatosis	57/133	71/131	X2 = 2.96; NS	51/109	77/154	X2 = 0.15; NS
Serum sclerostin (pmol/L)	40.41 ± 36.51 34.01 (17.47–54.08)	26.70 ± 22.78 17.02 (12.09–35.71)	*Z* = 2.64; *p* = 0.008	46.81 ± 42.77 38.32 (16.58 63.50)	29.66 ± 23.84 22.19 (14.20–39.53)	*Z* = 2.65; *p* = 0.008
Total cholesterol (mg/dL)	149.70 ± 49.49	164.62 ± 60.17	*T* = 2.32; *p* = 0.021	160.09 ± 56.09	155.15 ± 55.16	*T* = 0.74; NS
Triglycerides (mg/dL)	104.59 ± 47.60 93.0 (71.5–129.0)	134.53 ± 147.11 107 (76–148)	*Z* = 2.05; *p* = 0.040	118.16 ± 63.09 102 (77–140)	121.35 ± 134.73 100 (67–143)	*Z* = 0.98; NS
Serum creatinine (mg/dL)	0.92 ± 0.48 0.8 (0.62–1.08)	0.88 ± 0.55 0.8 (0.6–1.0)	*Z* = 1.23; NS	1.04 ± 0.60 0.9 (0.7–1.1)	0.81 ± 0.43 0,7 (0.6–0.9)	*Z* = 4.40; *p* < 0.001
Uric acid (mg/dL)	5.18 ± 3.40 4.5 (3.5–6.4)	5.13 ± 2.03 5.0 (3.7–6.6)	*Z* = 0.43; NS	5.73 ± 2.39 5.3 (3.9–7.5)	4.72 ± 1.95 4.4 (3.4–5.6)	*Z* = 3.14; *p* = 0.002
HbA1C (%)	5.99 ± 3.40 5.4 (5.0–5.9)	5.85 ± 1.51 5.5 (5.0–6.2)	*Z* = 1.15; NS	5.95 ± 1.42 5.5 (5.2–6.2)	5.96 ± 3.86 5.3 (4.9–5.7)	*Z* = 2.38; *p* = 0.017

Daily ethanol ingestion was greater among patients with vascular calcifications (*Z* = 2.18; *p* = 0.029). Duration of alcohol consumption was related to vascular calcifications (*Z* = 3.03; *p* = 0.002) but this relationship was displaced by age in the multivariate analysis.

No association was observed between vascular calcium deposits and liver cirrhosis (χ^2^ = 0.38; *p* = 0.54; NS). We also failed to find any relationship between prothrombin activity, serum bilirubin, or serum albumin (as variables related to liver failure) and calcium deposits, but calcium deposits were more frequently observed among Child A patients (χ^2^ = 7.00; *p* = 0.03), especially when only cirrhotics were considered (χ^2^ = 9.00; *p* = 0.011). Platelet count (*Z* = 2.34; *p* = 0.018; possibly related to portal hypertension) and serum GGT (*Z* = 2.56; *p* = 0.011), possibly related to ethanol consumption, were higher in patients with vascular calcium deposits, but not MCV (*Z* = 1.21; *p* = 0.23; NS).

Vascular calcium deposits were associated with obesity (BMI > 30; χ^2^ = 4.65; *p* = 0.031); 23% of patients with calcium deposits were obese vs. 11.71% of patients without calcium deposits. BMI was significantly higher among patients with calcium deposits than those without calcium deposits (*t* = 2.07; *p* = 0.039), a difference that kept a marginally statistical significance when patients with ascities were excluded (*Z* = 1.96; *p* = 0.05). Serum creatinine levels were non-significantly higher among patients with calcium deposits (0.92 ± 0.48 mg/dl) than among patients without calcium deposits (0.88 ± 0.55 mg/dl; *t* = 0.72; *p* = 0.47), and no association was observed among calcium deposits and chronic kidney failure (CKF, defined as serum creatinine in stable conditions >1.40 mg/dl; χ^2^ = 1.45; *p* = 0.23).

One hundred and seventeen patients (39.13%) were affected by hypertension, a proportion like to that observed in the control population (χ^2^ = 0.03; *p* = 0.86; NS, Table 1). A significant relationship was found between calcium in the X-ray plain film and hypertension (χ^2^ = 4.22; *p* = 0.04), although only 66 patients with vascular calcium deposits (46.48%) were diagnosed with hypertension.

Age was significantly higher among patients with vascular calcifications (*t* = 6.57; *p* < 0.001) and also among patients with hypertension (*t* = 5.49; *p* < 0.001). Hypertension was strongly associated with older age (*t* = 5.49; *p* < 0.0001), but it was not related to cirrhosis (χ^2^ = 0.07; *p* = 0.80; NS) or liver steatosis (χ^2^ = 0.57; *p* = 0.45; NS). Duration of alcohol consumption was related to hypertension (*Z* = 3.21; *p* = 0.001), but this relationship was displaced by age when this variable was also introduced in a multivariate analysis. A strong association was observed between hypertension and diabetes (*t* = 5.49; *p* < 0.001), between calcium deposits and diabetes (χ^2^ = 23.16; *p* < 0.001) and hypertension and obesity (χ^2^ = 13.96; *p* < 0.001). A total of 24 patients showed creatinine values (after stabilization) over 1.40 mg/dl. A strong association was observed among hypertension and CKF (χ^2^ = 9.11; *p* = 0.003).

Two hundred and two patients (68%) were also smokers. Tobacco consumption was not associated with hypertension (χ^2^ = 0.11; *p* = 0.74; NS) and showed a non-significant trend with the presence of vascular calcifications (χ^2^ = 2.85; *p* = 0.09). The index packets/year showed a trend to higher values among patients with vascular calcifications (*Z* = 1.75; *p* = 0.08) and was significantly higher among patients with hypertension (*Z* = 2.14; *p* = 0.033).

### Serum sclerostin and other factors related to the development of vascular calcifications

**FIGURE 2:**
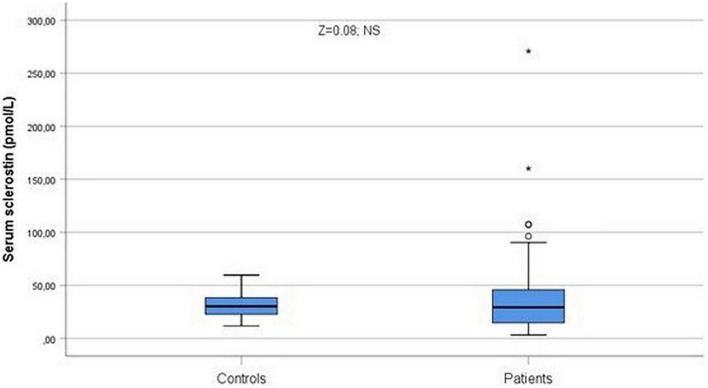
Serum sclerostin levels were slightly, non-significantly higher among patients (*Z* = 0.08; NS). °Represent the outliers and *represent the extreme outliers.

**FIGURE 3:**
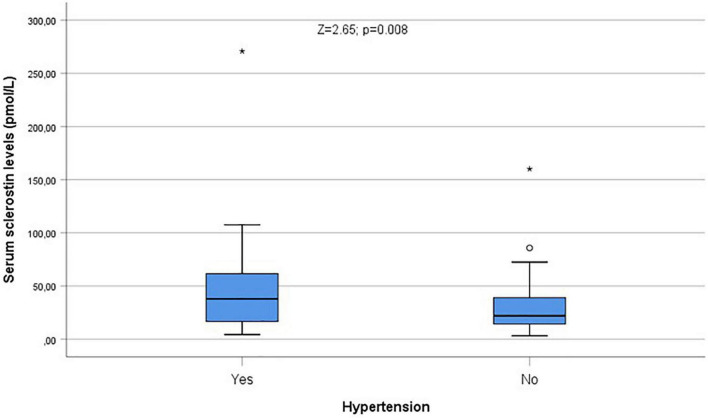
Serum sclerostin levels were higher in hypertensive patients (*Z* = 2.65; *p* = 0.008). °Represent the outliers and *represent the extreme outliers.

Serum sclerostin levels were slightly, non-significantly higher, among patients (Figure 2). Sclerostin levels were related to age (ρ = 0.30, *p* < 0.001), but no differences were observed among men and women (*Z* = 0.25; *p* = 0.80; NS). Patients with diabetes (*Z* = 2.10; *p* = 0.035) or hypertension (*Z* = 2.65; *p* = 0.008) showed higher sclerostin levels than patients without diabetes or hypertension (Figure 3). Cirrhotics showed non-significantly higher levels of sclerostin that non-cirrhotics (*Z* = 1.51; *p* = 0.13; NS).

**FIGURE 4:**
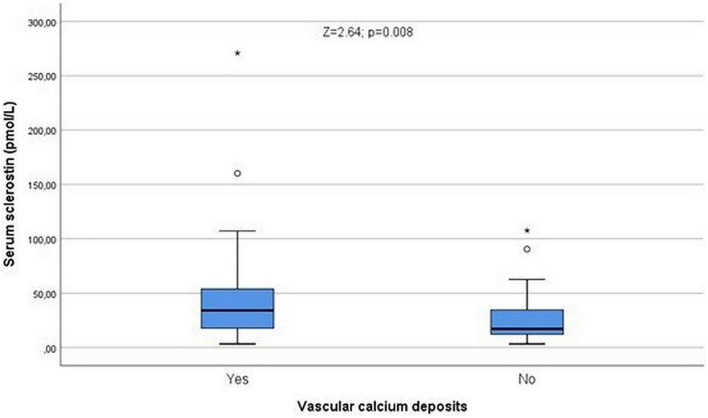
Serum sclerostin levels were higher in patients with vascular calcium deposits (*Z* = 2.64; *p* = 0.008). °Represent the outliers and *represent the extreme outliers.

Significant relationships were recorded comparing vascular calcifications with total cholesterol (*Z* = 2.04; *p* = 0.041) and triglycerides (*Z* = 2.05; *p* = 0.04). Sclerostin levels were significantly higher (*Z* = 2.64; *p* = 0.008, Figure 4) among patients with vascular calcifications, but not serum creatinine (*Z* = 1.23; *p* = 0.22; NS) or uric acid (*Z* = 0.43; *p* = 0.66; NS).

In summary, vascular calcifications are very frequent among alcohol misusers, and are related to age, diabetes, hypertension, sclerostin, cholesterol, triglycerides and daily ethanol intake, and marginally, to obesity assessed by BMI in patients without ascites. A logistic regression analysis including (as independent variables) sclerostin, total cholesterol, triglycerides (classified as dichotomic variables according to medians), CKF, cirrhosis, diabetes, and hypertension showed that sclerostin was the only variable independently related to the presence of vascular calcifications (*p* = 0.022; odds ratio for calcifications if sclerostin is over the median = 2.65 (95% CI = 1.14–6.13). However, this relationship was displaced by age if this variable was also included.

**FIGURE 5:**
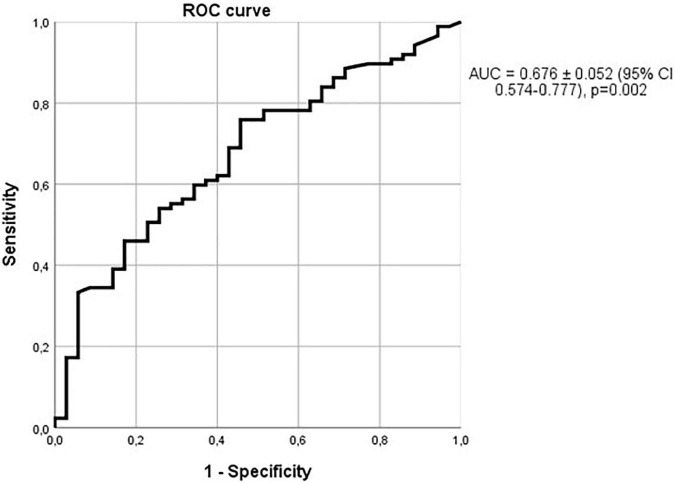
Relationship of sclerostin with vascular calcifications. ROC curve with an AUC of 0.676 ± 0.052 (95% CI 0.574–0.777); *p* = 0.002.

**FIGURE 6:**
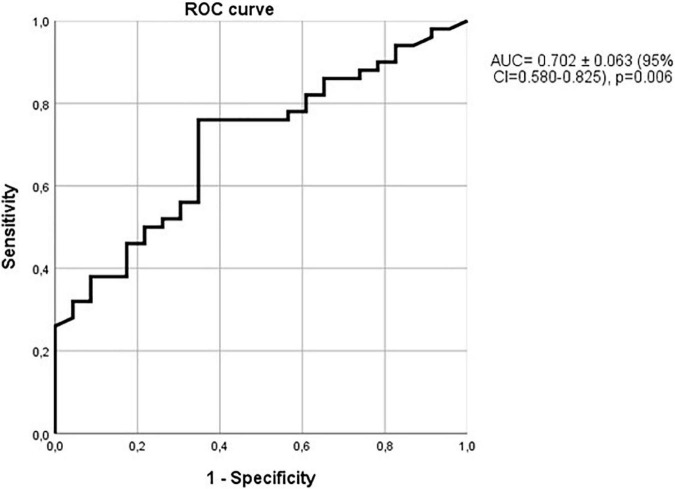
Relationship of sclerostin with vascular calcifications in non-hypertensive patients. ROC curve with an AUC = 0.702 ± 0.063 (95% CI = 0.580–0.825), *p* = 0.006.

The ability of sclerostin to diagnose vascular calcifications can be also observed with the ROC curve with an AUC of 0.676 ± 0.052 (95% CI 0.574–0.777; *p* = 0.002, Figure 5). As previously commented, only 66 of the patients with vascular calcifications were also affected by hypertension. Considering only the non-hypertensive patients, the relationship of sclerostin with vascular calcifications was even more marked, as shown in Figure 6, with an AUC of 0.702 (95% CI = 0.580–0.825), a standard error of 0.063 and a *p*-value of 0.006. Therefore, among alcoholics, sclerostin constitutes a risk factor for the development of vascular calcification, that, in our study, seems to be more important than the classic risk factors hypertension, kidney failure, cholesterol, or triglycerides.

### Brain atrophy

An expert radiologist evaluated the vast majority (296) CT studies and classified the patients as affected by cortical atrophy (218) or cerebellar atrophy (210), or not. As expected, CT indices were all significantly different among patients with cortical or cerebellar atrophy (Table 2) apart from Evans index, that was similar among patients with cerebellar atrophy and patients without cerebellar atrophy. A close association was observed among the presence of cerebellar atrophy and cortical atrophy (χ^2^ = 155; *p* < 0.001), although 12 patients with cerebellar atrophy (5.71%) did not show cortical atrophy, and 20 patients with cortical atrophy (9.17%) did not show cerebellar atrophy.

**TABLE 2:** Differences in CT indices in patients with or without cortical and cerebellar atrophy.

	Cortical atrophy			Cerebellar atrophy		
	Yes (*n* = 218)	No (*n* = 78)	T; p	Yes (*n* = 210)	No (*n* = 86)	T; p
Bicaudate index	0.18 ± 0.04	0.15 ± 0.03	*T* = 6.50; *p* < 0.001	0.18 ± 0.04	0.16 ± 0.04	*T* = 4.63; *p* < 0.001
Bifrontal index	0.35 ± 0.05 0.36 (0.33–0.38)	0.33 ± 0.05 0.33 (0.30–0.35)	*Z* = 4.89; *p* < 0.001	0.35 ± 0.05 0.35 (0.33–0.38)	0.33 ± 0.06 0.33 (0.30–0.36)	*Z* = 3.53; *p* < 0.001
Evans index	0.30 ± 0.04 0.30 (0.28–0.33)	0.29 ± 0.06 0.29 (0.26–0.30)	*Z* = 4.24; *p* < 0.001	0.30 ± 0.04 0.30 (0.28–0.33)	0.29 ± 0.06 0.29 (0.26–0.31)	*Z* = 3.17; *p* = 0.002
Ventricular index	0.54 ± 0.10 0.53 (0.47–0.60)	0.48 ± 0.11 0.46 (0.41–0.54)	*Z* = 4.45; *p* < 0.001	0.53 ± 0.10 0.52 (0.47–0.60)	0.50 ± 0.11 0.48 (0.42–0.57)	*Z* = 3.31; *p* = 0.001
Cella media index	4.02 ± 0.93 3.94 (3.49–4.42)	4.58 ± 1.09 4.52 (3.89–5.24)	*Z* = 4.42; *p* < 0.001	4.00 ± 0.91 3.93 (3.50–4.39)	4.60 ± 1.12 4.54 (3.90–5.24)	*Z* = 4.99; *p* < 0.001
Huckmann index	0.54 ± 0.08	0.48 ± 0.07	*T* = 5.65; *p* < 0.001	0.54 ± 0.07	0.49 ± 0.09	*T* = 3.97; *p* < 0.001

Age was significantly related to all the indices (Huckmann ρ = 0.34; Evans ρ = 0.29; Bifrontal ρ = 0.34, bicaudate ρ = 0.28; *p* < 0.001 in all the cases), ventricular ρ = 0.15, and cella media (ρ = 0.16; *p* < 0.01 in both cases). Duration of addiction was also significantly related to all the indices (Huckmann ρ = 0.29; Evans ρ = 0.23; Bifrontal ρ = 0.27, bicaudate ρ = 0.22; *p* < 0.001 in all the cases), and ventricular (ρ = 0.12) and cella media (ρ = 0.15; *p* < 0.033 in both cases), but these relationships were displaced by age when multivariate analyses were performed. No relationships were observed among CT indices and daily ethanol consumption.

**FIGURE 7:**
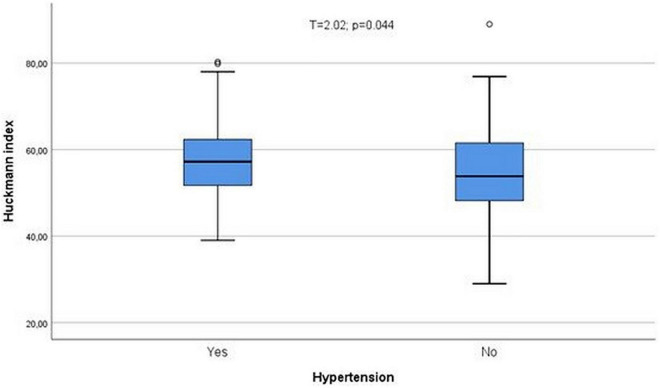
Patients with hypertension showed a greater atrophy estimated by Huckmann index (*T* = 2.02; *p* = 0.044). °Represent the outliers and *represent the extreme outliers.

No relationships were observed between obesity and brain CT indices, cirrhosis, and brain CT indices, or steatosis and brain CT indices. We also failed to find any relationship among CT indices and liver function assessed by Child-Pugh’s score. Patients with hypertension showed a greater atrophy estimated by Huckmann index (*T* = 2.02; *p* = 0.044, Figure 7). No differences were observed when the indices were compared among patients with or without CKF, besides cella media (*Z* = 1.97; *p* = 0.049), and no relationships were observed between CT indices and creatinine, besides a direct one between serum creatinine and ventricular index (ρ = 0.13; *p* = 0.03). All the indices besides Evan’s index were altered in diabetics (ventricular index *Z* = 3.16; *p* = 0.002; bicaudate index *Z* = 3.47; *p* = 0.001; bifrontal index *Z* = 2.07; *p* = 0.038; cella media *Z* = 1.98; *p* = 0.048; Huckmann index *Z* = 2.99; *p* = 0.003). All these differences were displaced by age (logistic regression analysis including diabetes, vascular calcifications, CKF, hypertension, and age, cholesterol and triglycerides, as dichotomic variables). However, diabetes still showed an independent relationship with Huckmann index, but in the second place, after age [OR = 2.28 (1.29–4.06; *p* = 0.005)].

**FIGURE 8:**
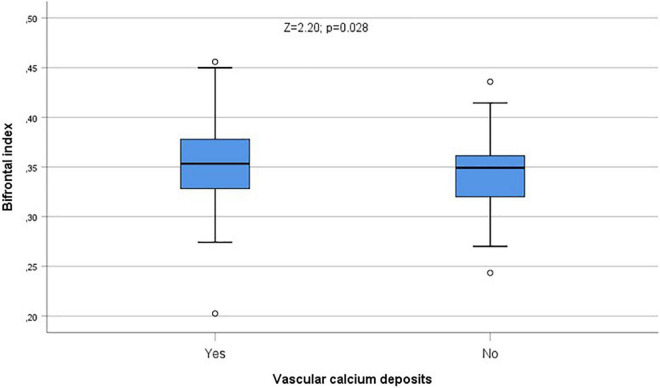
Calcium deposits were significantly related to Bifrontal index (*Z* = 2.20; *p* = 0.028). °Represent the outliers and *represent the extreme outliers.

**FIGURE 9:**
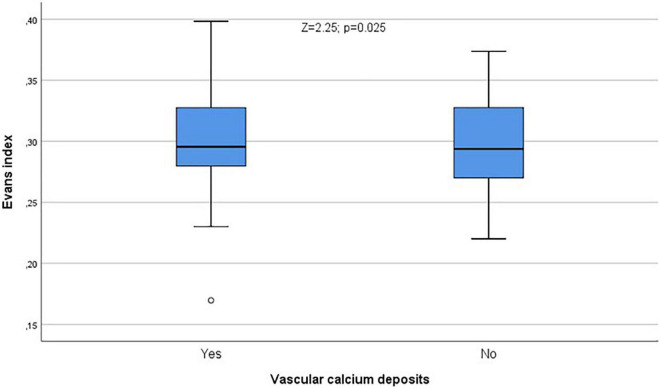
Calcium deposits were significantly related to Evans index (*Z* = 2.25; *p* = 0.025). °Rrepresent the outliers and *represent the extreme outliers.

In Table 3 we show the differences in CT indices in relation with vascular calcium deposits. Calcium deposits were significantly related to Bifrontal index (*Z* = 2.20; *p* = 0.028, Figure 8) and Evans index (*Z* = 2.25; *p* = 0.025, Figure 9), although these relationships were displaced by age in both cases when indices and age were classified as dichotomic variable and logistic regression analyses were performed comparing each of the CT indices as dependent variables with age and calcium deposits.

**TABLE 3:** Vascular calcium deposits and CT indices.

	Vascular calcium deposits		T (Z); p
	Yes (*n* = 143)	No (*n* = 152)	
Bicaudate index	0.17 ± 0.04	0.17 ± 0.04	*T* = 031; NS
Bifrontal index	0.35 ± 0.05 0.35 (0.33–0.38)	0.34 ± 0.06 0.34 (0.31–0.37)	*Z* = 2.20; *p* = 0.028
Evans index	0.30 ± 0.04 0.30 (0.28–0.33)	0.29 ± 0.05 0.29 (0.26–0.32)	*Z* = 2.25; *p* = 0.025
Ventricular index	0.52 ± 0.11 0.51 (0.45–0.57)	0.53 ± 0.10 0.51 (0.45–0.60)	*Z* = 1.08; NS
Cella media index	4.22 ± 0.98 4.13 (3.52–4.74)	4.14 ± 1.03 4.07 (3.62–4.53)	*Z* = 0.34; NS
Huckmann index	0.53 ± 0.08 0.53 (0.47–0.57)	0.51 ± 0.08 0.51 (0.46–0.56)	*T* = 1.38; NS

**FIGURE 10:**
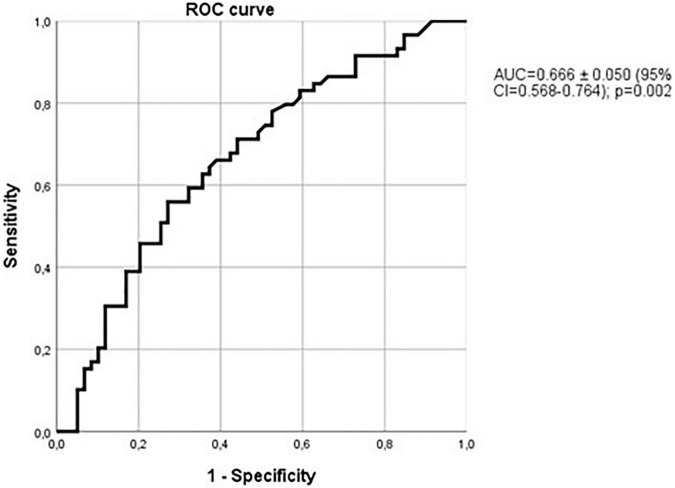
Relationship between sclerostin and cella media values over the median. ROC curve with an AUC = 0.666 ± 0.050 (95% CI = 0.568–0.764); *p* = 0.002.

Sclerostin levels showed a significant correlation with Huckmann index (ρ = 0.204; *p* = 0.024). In addition, cella media index was significantly different when patients with sclerostin values over the median were compared with patients with sclerostin values below the median (*Z* = 2.43; *p* = 0.015). The relationship between sclerostin and cella media index was also evident when a ROC curve was depicted in order to analyze sensitivity and specificity of sclerostin to detect patients with cella media values over the median, with an AUC of 0.666 ± 0.050 (95% CI = 0.568–0.764; *p* = 0.002, Figure 10).

**FIGURE 11:**
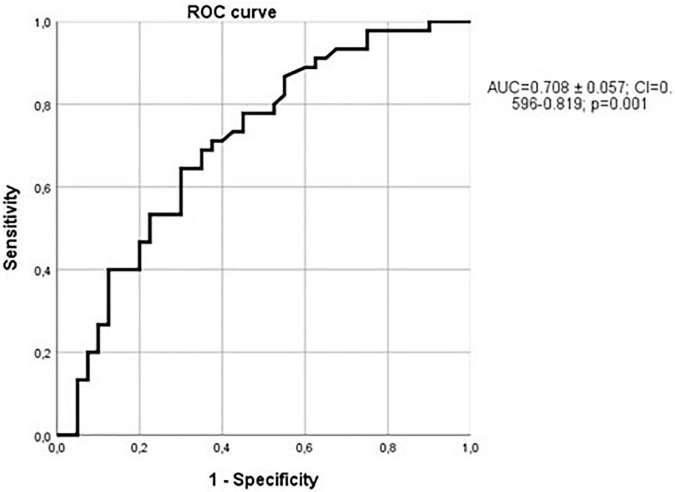
Relationship between sclerostin and cella media values over the median in patients with vascular calcifications. ROC curve with an AUC = 0.708 ± 0.057; (95% CI = 0.596–0.819); *p* = 0.001.

A stepwise logistic regression analysis comparing cella media values over the median (as the dependent variable) with sclerostin, cholesterol, triglycerides, daily ethanol consumption (as dichotomic variables according to median values), vascular calcifications, hypertension, and CKF, showed that sclerostin (over the median) was the only variable selected (odds ratio = 2.5, 95% confidence interval = 1.16–5.39; *p* = 0.019), and the same happened when the variable age (dichotomized) was also introduced. Considering only patients without vascular calcification, the ROC curve comparing sclerostin with the cella media index over or below the median was not significant at all (AUC = 0.518 ± 0.105; *p* = 0.86), in contrast with what was observed when only patients with vascular calcifications were included (AUC = 0.708 ± 0.057; 95% CI = 0.596–0.819; *p* = 0.001, Figure 11). Therefore, the relationship of sclerostin with brain atrophy is especially marked in patients with vascular calcifications.

**FIGURE 12:**
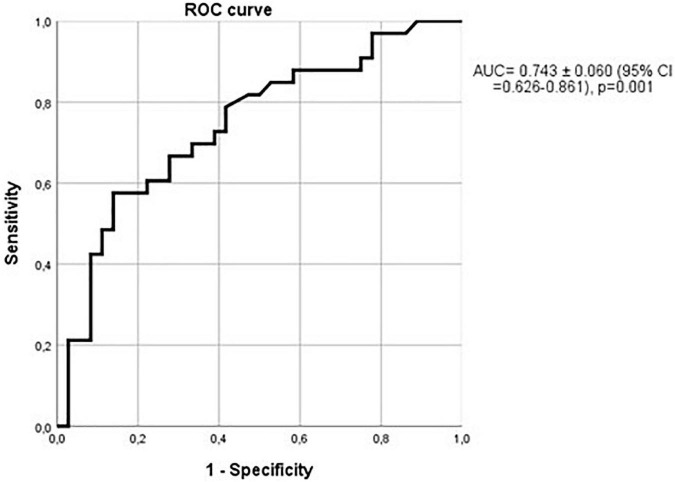
Relationship between sclerostin and cella media values over the median in non-hypertensive patients. ROC curve with an AUC = 0.743 ± 0.060 (95% CI = 0.626–0.861); *p* = 0.001.

In hypertensive patients, the ROC curve comparing sclerostin and cella media index was not statistically significant (AUC = 0.588 ± 0.095; 95% CI = 0.401–0.775; *p* = 0.31), but considering only non-hypertensive patients, ROC curve analysis yielded an AUC even greater [0.743 ± 0.060 (95% CI = 0.626–0.861; *p* = 0.001)] than that observed in the whole group, as shown in the Figure 12. Therefore, when hypertension is present, the relationship of sclerostin with brain atrophy is less marked.

**FIGURE 13:**
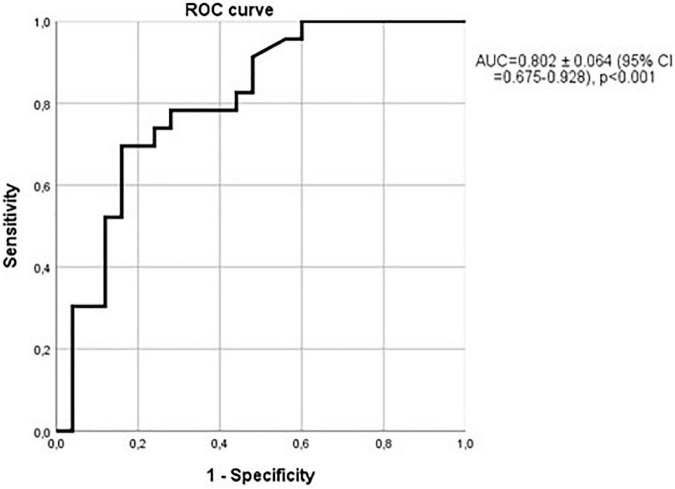
Relationship between sclerostin and cella media values over the median in non-hypertensive patients with vessel wall calcium deposits. AUC = 0.802 ± 0.064; (95% CI = 0.675–0.928); *p* < 0.001.

Lastly, the ability of sclerostin in the diagnosis of brain atrophy (cella media index) is even more marked in non-hypertensive patients with vessel wall calcium deposits. AUC reaches 0.802 ± 0.064; (95% CI = 0.675–0.928; *p* < 0.001; Figure 13).

Also, a stepwise logistic regression analysis within no-hypertensive patients, comparing cella media values over the median (as the dependent variable) with sclerostin, cholesterol, triglycerides, daily ethanol consumption (as dichotomic variables according to median values), vascular calcifications, and CKF, showed that sclerostin (over the median) was the only variable selected (odds ratio = 2.93; 95% CI = 1.08–7.94; *p* = 0.035), and the same happened when the variable age (dichotomized) and/or diabetes were also introduced.

Therefore, it seems that sclerostin also constitutes a risk factor for subcortical brain atrophy (assessed by cella media index), both in the global alcoholic population and in non-hypertensive patients, independent of age and other common risk factors such as vascular calcifications, cholesterol, triglycerides, or diabetes.

## Discussion

In this study we estimated the prevalence of vascular calcifications in the aortic arch as a marker of vascular injury, and the relationship of this feature with brain atrophy in alcoholics. We also pursued to analyze the relationship of an emerging new vascular risk factor, namely sclerostin with these alterations.

We found that the prevalence of vascular calcifications is very high among alcoholic patients, and vascular calcifications are significantly related to brain shrinkage in these patients. Although in an observational study like this we cannot establish a causal link between both features (brain shrinkage and calcium vessel wall deposits), our results suggest that vascular damage may be a contributory non-functional, but organic, factor involved in the brain alterations observed in these patients.

The relationship of vascular calcifications with chronic ethanol intake has been pointed out in previous studies. For instance, the relationship between coronary artery calcification and ethanol consumption has been recorded by several authors (Pletcher et al., 2005; Yun et al., 2017), who also report an increased prevalence of hypertension in excessive drinkers, in accordance with former observations (Saunders, 1987; van Leer et al., 1994; Fuchs et al., 2001). However, in this study, prevalence of hypertension among alcoholics is similar to that of the controls, and also similar to that reported for the general population of our geographical environment (Cabrera De León et al., 2008; Zubeldia Lauzurica et al., 2016). The markedly higher prevalence of vascular calcification among alcoholics, despite a similar prevalence of hypertension in patients and controls, strongly suggests that factors other than hypertension should play a role in the calcification of vessel walls.

As previously commented. ethanol may exert direct effects on smooth muscle cells of the vessel walls, promoting vascular smooth muscle cells calcification and transition of these cells to osteoblastic-like cells (Oros et al., 2012). Therefore, radiologically detectable vascular calcifications may be observed in excessive drinkers, independent of the concomitant presence of hypertension. Oros’ research also lends support to the findings of this study, in which calcium deposits were recorded in 145 (48%) excessive drinkers, but only 66 out of 145 patients with vascular calcifications were also affected by hypertension, suggesting that other factors also play a pathogenetic role.

In this study we tested the relationship of sclerostin both with vascular damage and brain atrophy in the whole sample, considering together hypertensive and non-hypertensive patients, and also in the subgroups of hypertensive and non-hypertensive patients, these last lacking a classic major risk factor for vascular damage. As commented in the introduction of this study, sclerostin may favor calcification of vessel walls (Zhu et al., 2011), atherosclerotic plaques (Leto et al., 2019), and aortic valves (Koos et al., 2013). We found that sclerostin levels were higher among patients with vascular calcifications. Therefore, our results are in accordance with these observations. The fact that sclerostin is the sole independent factor related to vascular calcification in a logistic regression analysis, being displaced only by age, is a striking result. Of similar importance is the finding observed in non-hypertensive patients, in whom the relation of sclerostin with vascular calcification is even stronger. This may suggest that, at least in alcoholics, sclerostin may be a factor related to vascular damage, independent on hypertension and/or kidney failure (a suggestion also derived from the reported results of the logistic regression analysis).

Importantly, sclerostin levels were not only related to vascular calcification in our study, but also to a possible consequence of vascular calcification, namely brain atrophy. In addition to the already commented many factors involved in the development of brain atrophy in alcoholics, the role of altered vascular supply in alcoholics has been also pointed out, not only by the commented relationship between hypertension and alcoholism, but also as an additional direct functional effect of ethanol. Since several decades, ethanol is known to cause cerebral arterial spasm, bleeding, and alterations of blood flow in certain areas of the brain (Altura and Altura, 1984), such as temporal, frontal and occipital cortices, corpus callosum, and basal ganglia. These effects, together with hypertension, may explain the association of ethanol consumption and stroke (Schuckit, 2009), but also, possibly, the association of ethanol with brain atrophy and cognitive impairment. In this sense, we found a clear-cut relationship between calcium deposits in the thoracic vessel walls and brain atrophy, suggesting a role of vascular lesions on ethanol-mediated brain alterations. As expected, age was the main factor responsible for vascular calcifications and brain atrophy, but it is important to remark the independent relationship of sclerostin with vessel wall calcification and brain alterations (even displacing age), results that are in accordance with the current consideration of sclerostin as a major vascular risk factor. Strikingly, the best relationship with brain atrophy (assessed by cella media index) was observed among non-hypertensive patients with vascular calcifications. Taken together the results of the ROC analyses and the logistic regression analyses, it could be hypothesized that, in absence of hypertension as a major risk factor, the role of sclerostin on brain atrophy gains prominence probably by inducing vessel wall calcification, perhaps sharing or potentiating the effects of ethanol. This potentiating effect is a speculative possibility supported by the observation that the control group of non-drinkers show a very low proportion of vascular calcifications despite a similar prevalence of hypertension and similar sclerostin levels than the patients. Future research devoted to disentangling these possible connections is needed.

Brain damage in alcoholics affects both grey matter and white matter (de La Monte and Kril, 2014), with neuronal shrinkage and loss of dendritic spines, as well as axonal damage, atrophy, and ventricular dilatation. One of the most striking features of alcohol-mediated brain shrinkage is that it in many patients it is almost fully reversible with alcohol withdrawal. Therefore, transient, reversible, metabolic alterations, timely related to heavy alcohol consumption, probably play a major role in brain atrophy in these patients. Perhaps sclerostin is one of these factors, its deleterious effects on vascular structure being triggered by heavy alcohol consumption. In any case, the findings of this study do not support any role of ethanol (at least, in heavy consumers) as a “protective” vascular factor.

One limitation of this study resides in the fact that it was devoted to analyzing radiographically assessed alterations of brain and vascular lesions in alcoholics, but it did not include a functional evaluation. However, previous reports have shown a definite relationship between brain atrophy and/or ventricular dilatation and cognitive impairment in these patients, so brain atrophy can be considered as the structural alteration underlying the functional derangement (de La Monte and Kril, 2014). Many confounding factors, not assessed in this study, such as altered levels of vitamins, micronutrients, and aspects related to style of life and education may be surely involved in the brain alterations of alcoholic patients (as well as in non-alcoholics). Possibly, their correction may contribute to the reversible nature of brain damage and cognitive impairment of alcoholics after drinking cessation, although this study strongly suggest that potentially irreversible vascular lesions may play a contributory role in brain atrophy; and also, perhaps, that ethanol may interact with molecules involved in vessel wall metabolism, such as sclerostin, potentiating its deleterious effects.

## Conclusion

We conclude that prevalence of vascular calcification in alcoholics is very high, despite the relatively young age of the included individuals. Among nearly 300 individuals, vascular calcium deposits were strongly related to brain atrophy. Interestingly in addition to age, sclerostin was strongly related to vascular calcifications, and it was also independently related to brain atrophy, underscoring the role of osteokines on vascular disorders, at least in excessive drinkers.

## Data availability statement

The original contributions presented in this study are included in the article/supplementary material, further inquiries can be directed to the corresponding author.

## Ethics statement

The studies involving human participants were reviewed and approved by Institutional Ethics Committee of Hospital Universitario de Canarias, and all subjects provided informed written consent (Approval number: 2017_50). The patients/participants provided their written informed consent to participate in this study.

## Author contributions

CM-G and EG-R contributed to the conceptualization, writing—review and editing, formal analysis, and writing—original draft. AG-R, CF-R, EM-P, MS-P, JA-N, and MR-G contributed to the investigation. PA-G contributed to the methodology. All authors contributed to the article and approved the submitted version.
